# Application of Diamond Nanoparticles in Low-Energy Neutron Physics

**DOI:** 10.3390/ma3031768

**Published:** 2010-03-10

**Authors:** Valery Nesvizhevsky, Robert Cubitt, Egor Lychagin, Alexei Muzychka, Grigory Nekhaev, Guillaume Pignol, Konstantin Protasov, Alexander Strelkov

**Affiliations:** 1 Institut Laue-Langevin, 6 rue Jules Horowitz, Grenoble, F-38046, France; E-Mail: cubitt@ill.fr (R.C.); 2 Joint Institute for Nuclear Research, 6 Joliot Curie, Dubna, Moscow reg., 141980, Russia; E-Mails: lychag@nf.jinr.ru (E.L.); muz@nf.jinr.ru (A.M.); str@jinr.ru (A.S.); 3 Laboratoire de Physique Subatomique et de Cosmologie, UJF Grenoble 1, CNRS/IN2P3, Grenoble INP, 53 rue des Martyrs, Grenoble, F-38026, France; E-Mails: guillaume.pignol@polytechnique.org (G.P.); protasov@lpsc.in2p3.fr (K.P.); 4 Research Institute of Materials Technology, Presnenskii val, 21/18, Moscow, 123557, Russia

**Keywords:** diamond nanoparticles, slow neutrons, neutron albedo

## Abstract

Diamond, with its exceptionally high optical nuclear potential and low absorption cross-section, is a unique material for a series of applications in VCN (very cold neutron) physics and techniques. In particular, powder of diamond nanoparticles provides the best reflector for neutrons in the complete VCN energy range. It allowed also the first observation of quasi-specular reflection of cold neutrons (CN) from disordered medium. Effective critical velocity for such a quasi-specular reflection is higher than that for the best super-mirror. Nano-diamonds survive in high radiation fluxes; therefore they could be used, under certain conditions, in the vicinity of intense neutron sources.

## 1. Introduction

In this short communication we present the most interesting results on applications of detonation nano-diamonds in low-energy neutron physics. The formation of diamond nanoparticles by explosive shock was first observed nearly 50 years ago [1]. Since then, very intensive studies of their production and various applications have been performed worldwide. These particles measure a few nanometers. They consist of a diamond nucleus (with a typical diamond density and optical nuclear potential) within an onion-like shell of a complex chemical composition [2] (with significantly lower optical potential). A recent review of the synthesis, structure, properties and applications of diamond nanoparticles can be found, for instance, in ref. [3]. Such detonation nano-diamonds are very attractive for various applications in low-energy neutron physics due to their several unique properties. Thus, the critical velocity of diamond, 8 m/s, is the highest available, thus providing the broadest spectrum of reflected ultracold neutrons (UCN), and the highest scattering efficiency for VCN (very cold neutrons) and for CN (cold neutrons). The absorption cross-section of carbon is exceptionally low, ~3.5 mb, thus providing a huge number of consecutive scattering events. This last point is particularly important for interference phenomena (as Anderson localization, for instance) as well as for long diffusion motion of slow neutrons in powders of diamond nanoparticles. In fact, neutron losses are dominated by impurities on the surface of nanoparticles, in particular by small impurity of hydrogen with its huge inelastic cross-section (up to ~10^2^ b) and significant absorption cross-section (0.3 b), even at zero temperature. That is why proper control/elimination/substitution of hydrogen is a major issue in any neutron application of nano-diamonds; on the other hand, neutrons provide an extraordinary probe to study hydrogen in the powders. 

Let’s remember the basic features and applications of slow neutrons. UCN are neutrons with very low energy of smaller than a few times 10^-7^ eV. They are totally reflected from surface at any incidence angle if their energy is lower than the effective nuclear optical potential of surface material [4,5,6,7]. The characteristic penetration depth is close to its wavelength and equal to a few tens of nanometers. VCN with typical energy of 10^-7^−10^-4^ eV and CN with typical energy 10^-4^−10^-2^ eV are totally reflected from a flat surface only if the incidence angle is sufficiently small; so that the perpendicular-to-surface component of the neutron velocity is lower than the material critical velocity. Slow neutrons, in particular UCN and CN, provide an excellent tool for various highly sensitive experiments in fundamental neutron physics: for instance, searches for a non-zero electric dipole moment of the neutron [8,9], searches for a non-zero electric charge of the neutron [10], and various experiments using gravitationally bound quantum states of neutrons [11]. However, the low density/flux of UCN available is a severe limiting factor. If experiments are limited by systematic uncertainties (such as the precision measurements of the neutron lifetime [12,13,14,15,16,17]), high UCN density is also useful, because it allows one to reveal easier systematic effects. Besides that, high UCN density plays a decisive role in any application of UCN in physics of surface or nanoparticles [18,19]. That is why many laboratories devote significant efforts to increase the UCN density/flux. The method of liquid or solid converters has been well developed during the last decades [7,20,21,22,23,24,25,26,27,28]. Down-scattering of neutrons in liquid ^4^He was investigated as well [7,29,30,31]. A method of equilibrium neutron thermalization at ultracold nanoparticles was proposed [18]. For all the mentioned methods, the neutron transport from a UCN source to an experimental installation is of critical importance (see, for instance, Refs. [24,32,33,34]). CN is the best tool for precision studies of the neutron β-decay [35,36]. They are intensively used in studies of fundamental symmetries [37,38]. However, the intermediate energy range of VCN has not yet been intensively explored in this domain, except for a few measurements, for instance, [39]. On the other hand, any progress in the mentioned above experiments followed from preceding methodical and technological developments. We hope for a break-through in use of VCN in this field due to recent results on VCN scattering on diamond nanoparticles described here.

Generally speaking, the reason for a significant interplay between slow neutron physics on one hand, and the nanoparticle physics on the other hand, consists in an approximate equality of parameters of neutrons and nanoparticles under certain conditions [18]. The neutron wavelength λ/2π could be close to the nanoparticle radius r. Simultaneously, the maximum neutron energy increase at a single collision event with a free (or weakly bound) nanoparticle could be close to the neutron initial energy, as clear from the following condition: in terms of the neutron velocity V and the nanoparticle thermal motion velocity V_T_ this means (V + 2V_T_)^2^ = 2V^2^, or V ≈ 5V_T_. This condition is satisfied at ambient temperature for a typical nanoparticle radius of ~ 5 nm. 

If size of an object is compatible to the neutron wavelength, one rather discusses scattering but not reflection of neutrons; this is typically the case of scattering of slow neutrons on nanoparticles. A theoretical analysis of such scattering in the first Born approximation could be found, for instance, in ref. (40). The scattering amplitude on a single round particle with uniform optical potential equals

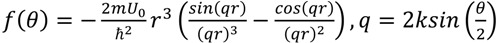
(1)
where *θ* is the scattering angle, *m* is the neutron mass, *U*_0_ is the real part of the nanoparticle optical potential, *ћ* is the Planck constant, *r* is the nanoparticle radius, $k=\frac{2\pi }{\lambda }$ is the neutron wave vector, and *λ* is the neutron wavelength. The scattering cross-section equals

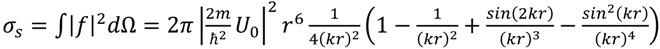
(2)
the cross-section of absorption of neutrons in a nanoparticle with the imaginary part of the neutron-nuclei optical potential *U*_1_, equals

(3) $$\sigma_{a}=\frac{4\pi }{3}\frac{2m}{\hbar^{2}}U_{1}r^{4}\frac{1}{kr}$$

These simple formulas are usually sufficiently precise to describe qualitatively all phenomena of interest. Interference between scatterings at neighbor nanoparticles could be neglected if the neutron velocity is not too low. Mie scattering [41] of neutrons by single nanoparticles to very large angles could be neglected for not too narrow distributions of neutron velocities and nanoparticle sizes.

Section 2 describes results of reflection of VCN from powders of nanoparticles. Section 3 deals with quasi-specular reflection of faster neutrons (CN) from powders of nanoparticles at above-critical angles. Finally, section 4 presents the possibility of studying motions of nanoparticles on surfaces using UCN. In all the considered cases, a new quality in the interaction of slow neutrons with medium appears due to the approximate equality of the neutron wavelength and the nanoparticle size, and/or on approximate equality of their velocities. The aim of this paper is to give a summary of applications of detonation nano-diamonds in low-energy neutron physics.

## 2. Reflection of VCN from Powder of Diamond Nanoparticles

Scattering of waves and particles on interfaces of disordered media represents an important topic in different domains of science [42]. Thus, the interaction of electromagnetic waves with atmospheric inhomogeneities, ground relief, ocean, aerosols, rain, snow, biological issues, composite materials, and other media is overviewed in ref. [43]. Charged particles (protons, electrons) or neutral particles (neutrons, atoms) provide another example of wave scattering on interfaces of disordered media. The principle condition that defines the domain of wavelengths of interest is approximate equality of the particle wavelength and the scatterer size. The first experiments on scattering of VCN from nano-structured materials as well as on VCN storage were carried out in the seventies in ref. [44] and were later continued in ref. [45]. However, until recently, albedo of neutrons from disordered medium had not been intensively studied/used because the effects observed were not significant. 

Things changed since one started using diamond nanoparticles due to fortunate coincidence of several of their parameters: the exceptionally high value of the optical potential of diamond, low neutron losses in powders of diamond nanoparticles, and the availability of the nanoparticles in optimum sizes (approximate equality of the neutron wavelength and the nanoparticle size of ~5 nm). These properties allowed one to efficiently reflect VCN on powders of diamond nanoparticles [46], thus bridging the energy gap between efficient reactor reflectors [47] for thermal and cold neutrons and optical neutron-matter potential for UCN. The use of nanoparticles provides a sufficiently large cross-section of coherent interaction and inhomogeneity of the reflector density on a spatial scale of about the neutron wavelength [18]. A large number of diffusive neutron-nanoparticle collisions needed to reflect VCN from powder constrains the choice of materials: only low absorbing materials with high optical potential are appropriate. Thus, diamond nanoparticles are an obvious choice for VCN reflector. 

Consider an idealized case of reflection of a neutron from an infinitely-thick loss-free powder of nanoparticles occupying half-space. After multiple scattering events, the neutron returns to the surface. In the case of non-zero imaginary part of the neutron-nuclei optical potential, finite absorption in nanoparticles decreases reflectivity. Nevertheless, neutrons with some wave vector are efficiently reflected. A reflector of this type would be particularly useful for both UCN sources using ultracold nanoparticles [18,40] and for VCN sources; it would not be efficient however for cold and thermal neutrons, as shown in ref. [48]. The VCN storage allows us to accumulate a significant number (density) of VCN in a trap (much larger than that typical for UCN). General feasibility of such a nano-powder reflector was verified in ref. [46]. In order to measure precisely the VCN reflection probability from powder of diamond nanoparticles and to explore feasibility of VCN storage in traps with nano-structured walls, we carried out a dedicated study [49]. 

**Figure 1:**
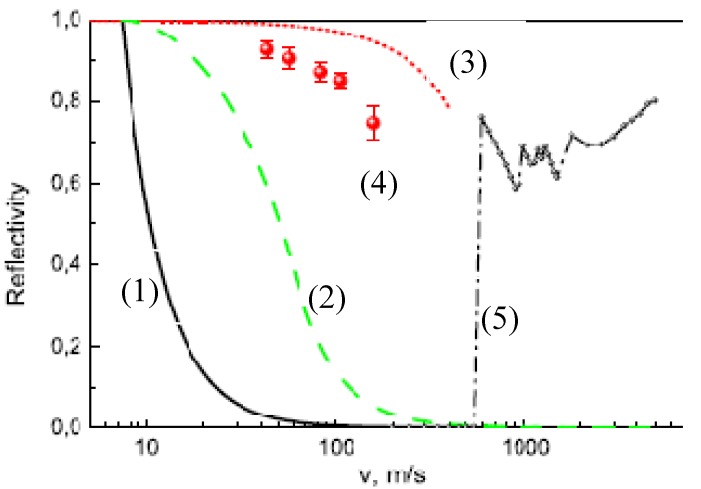
The elastic reflection probability for isotropic neutron flux is shown as a function of the neutron velocity for various carbon-based reflectors: (1) Diamond-like coating (DLC) (thin solid line), (2) The best supermirror [50] (dashed line), (3) Hydrogen-free ultradiamond powder with the infinite thickness (dotted line). Calculation. (4) VCN reflection from 3 *cm* thick diamond nano-powder at ambient temperature (points), with significant hydrogen contamination [49]. Experiment. (5) MCNP calculation for reactor graphite reflector [47] with the infinite thickness at ambient temperature (dashed-dotted line).

The probability of neutron isotropic flux reflection from diamond nanoparticles is compared with other known reflectors in Figure 1. Evidently, the maximum energy of the reflected VCN and the reflection probability far exceed the corresponding values for the best supermirror available [50]. Further improvement of the VCN storage times could be achieved by removing a part of hydrogen from powder, by isotopic substitution of hydrogen by deuterium, or/and by cooling a trap to a temperature, at which the inelastic up-scattering of VCN at hydrogen is strongly suppressed. 

## 3. Quasi-Specular Reflection of Cold Neutrons from Nano-Dispersed Media at Above-Critical Angles

Now, let’s consider faster neutrons so that λ « r. If so, the angle of neutron scattering on each nanoparticle is small. Therefore, neutrons arriving at a large incidence angle penetrate too deep into the powder and do not return to the surface before they are absorbed. Neutrons arriving at a small incidence angle α could return to surface after several small-angle scattering events. Such a neutron albedo is analogous to the process considered in a general form in ref. [51], where an analytic expression describing the angular spectrum of reflected radiation is found for various laws of single scattering of ions, electrons, protons and photons from a medium consisting of scattering centers with sizes significantly larger than the radiation wavelength. As the typical number of scattering events is small, the exit angle β is not much higher than α. In addition, the penetration depth and path of neutrons in the powder are small; therefore the absorption affects reflectivity much less than that in the previous case.

In fact, for the problem parameters used in the present study, the most probable exit angle β is approximately equal to the incidence angle α, with a diffusive halo around this angle. We call such a process quasi-specular reflection. Note that coherent scattering of neutrons from neighbouring nanoparticles is neglected here as it is small for the considered case λ « r. Besides, we neglected Mie scattering of neutrons (analogous to [41]), which assumes—in contrast to that in the first Born approximation—deviation of some neutrons to very large angles. Our simplification is justified as far as we are interested in the dominant small angle scattering.

**Figure 2:**
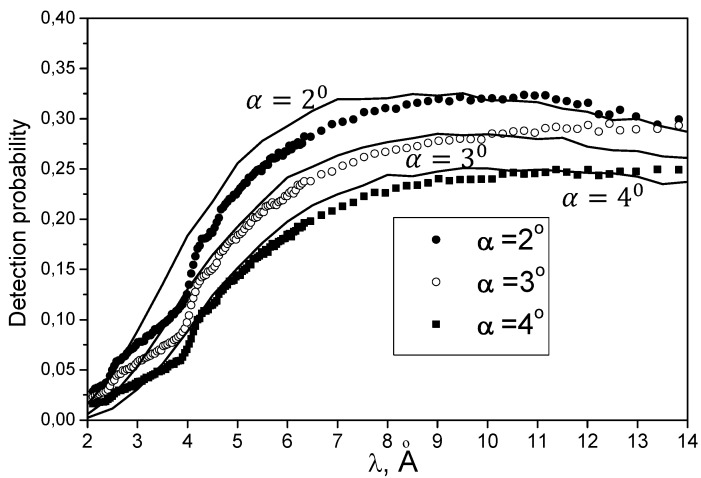
The probability of neutron reflection within the detector solid angle is shown as a function of the neutron wavelength; corresponding to 40–50% total probability (albedo) of quasi-elastically scattered neutrons. The incidence angle α is equal to 2°, 3°, and 4°. Dark and empty circles as well as squares correspond to measured data; solid lines illustrate calculations.

**Figure 3:**
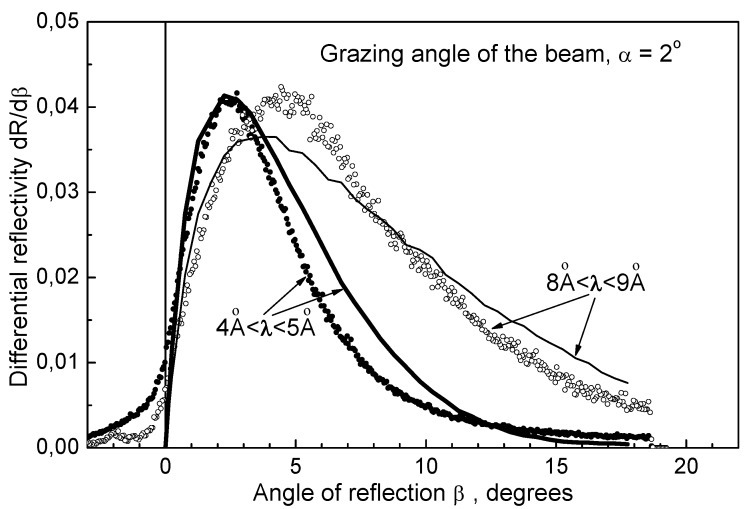
Angular distributions of reflected neutrons. The incidence angle is equal 2°. Dark and empty circles correspond to measured data; solid lines illustrate calculations.

Results of a measurement [52] are shown in Figure 2 and Figure 3.

Figure 2 shows the probability of neutron reflection within the detector solid angle as a function of the neutron wavelength and the incidence angle. The reflectivity values in Figure 2 are smaller than actual ones by a fraction of neutrons scattered to angles larger than the detector solid angle. Results of measurements at ambient and nitrogen temperature do not differ significantly. In particular, this is due to the small number of scattering events involved in quasi-specular reflection. The temperature-dependent inelastic neutron scattering is small (σ_in_(2200 m/s) = 1 b) compared to temperature-dependent elastic cross-section (σ_el_ = 120 b) and a fraction of hydrogen atoms in annealed nanoparticles is low: 1/15. Moreover, hydrogen is strongly bound to carbon; the phonon excitation spectrum is close to that for diamond. Any neutron scattering at hydrogen (both elastic and inelastic ones) is isotropic, therefore such a scattered neutron is almost totally lost. We estimate neutron losses at hydrogen to be equal to 20–40% for various incident angles α actually used. The wavelength range of effective quasi-specular reflection is limited to below ~4 Å by Bragg scattering of neutrons in the bulk of a diamond nanoparticle.

Computer simulation of quasi-specular reflection is straightforward; it is based on formulas (1–3). 

Figure 3 shows measured (dark and empty circles) and calculated (solid lines) angular distributions of reflected neutrons; the neutron incidence angle is 2°. The data are averaged over two ranges of wavelengths of incident neutrons: 4–5 Å, and 8–9 Å. The neutron count rates in the position-sensitive detector for every deviation angle are divided by the incident neutron flux at the same wavelengths. Some broadening of the calculated angular distributions compared to the measured data is explained by the simplification of the model (equal sizes of nanoparticles). Nevertheless, the general agreement of the data and such a simple model are quite good. 

Thus, quasi-specular reflection of cold neutrons from a nano-dispersed medium occurs at small incidence angles due to several small-angle scattering of neutrons at the nano-sized inhomogeneity of the effective neutron-nuclei potential. In contrast to standard sub-critical reflection of neutrons from optical potential of uniform medium, the quasi-specular reflection might be observed also at highly above-critical angles. Moreover, powders of diamond nanoparticles used here reflect neutrons with perpendicular velocity components larger than 40 m/s, the cut-off for the best super-mirrors available [50]. Quasi-specular reflection could find numerous applications, in particular, for neutron reflectors in zones close to a reactor core, where other reflectors would not survive radiation damage. These reflectors would increase significantly the flux of cold neutrons available for experiments. 

## 4. Motions of Nanoparticles at Surface—Quasi-Elastic Reflection of UCN

Until recently, the traditional description of UCN interaction with matter had assumed their loss from closed traps *via* three channels: their β-decay, their absorption by nuclei in the matter of trap walls, and their inelastic scattering on trap walls. This third possibility assumed the most probable scattering of UCN to the energy range corresponding to the wall temperature; this value exceeds the kinetic energy of the UCN by five orders of magnitude. However, an additional mechanism for UCN loss from traps was discovered. The UCN energy increased by ~10^-7^ eV with the probability of ~10^-8^–10^-5^ per collision [53]; this value exceeded theoretical expectations by many orders of magnitude. If the neutron energy after such inelastic scattering exceeds some critical value it would escape from the trap. This process is similar to the vaporization of UCN from a trap. The small heating of UCN has been studied over the last years both on solid surfaces (stainless steel, copper, beryllium *etc*.) and on liquid surfaces (different kinds of hydrogen-free oils) [53,54,55,56,57,58,59,60,61,62,63]. The experimental discovery of the small heating of UCN required a revision of our theories on the interaction of UCN with a surface [64] and a careful consideration of the different processes at the surface capable of providing inelastic scattering of UCN summarized in refs. [65,18]: only the scattering of UCN at clusters with a size of ~ 10nm can explain the experimental data obtained; the velocity of the clusters has to correspond to the observed values of energy change. Although the size distribution of nanoparticles and their compact conglomerates could be broad, neutrons are able to “select” nanoparticles with the size close to UCN wavelength irrespective of the particular distribution of nanoparticle sizes. 

The experimental methods used for this study and the experimental set-up are described in detail in [66] and in references herein. The principle of observation could be understood as follows. A typical UCN energy of ~10^-7^ eV corresponds to falling down of UCN from the height of ~1m in the earth’s gravitational field; thus UCN motion is strongly affected by gravity. This is why UCN could be confined by the gravitational barrier and their energy spectrum could be shaped from above by an absorber installed at definite height. If the energy of UCN increases due its interaction with a sample (or with a spectrometer wall), such UCN might leave the volume confined by the gravitational barrier and could be detected. Extremely intensive small heating of UCN on weakly bound diamond nanoparticles on a surface in a state of thermal motion was observed using this method. On one hand, these observations are a major step-forward in understanding of UCN interaction with surface. On the other hand, they provide a unique method to study dynamic of nanoparticles and nanostructures, a use hitherto considered impossible. 

For completeness of this short overview, we have to mention rapidly growing applications of diamond-like-coatings (DLC) for UCN vessels. Analogous to advantages of diamond nanoparticles, DLC provide the highest critical velocity and low UCN losses [67,68,69,70,71,72,73,74,75]. DLC coating using IBS technique (ion beam sputtering) with extremely small surface roughness on a flat surface could be even used for long storage of UCN at specular trajectories [76].

## 5. Conclusions

Diamond, with its exceptionally high optical nuclear potential and low absorption cross-section, is a unique material for a series of applications in VCN physics and techniques. In particular, powder of diamond nanoparticles provided the best reflectors for neutrons in the complete VCN energy range, and allowed the first observation of quasi-specular reflection of cold neutrons from disordered medium. Nano-diamonds survive in high radiation fluxes; therefore they could be used in vicinity of intense neutron sources, where other solutions are ruled out. In the energy range of UCN, diamond nanoparticles triggered unique investigations of motions of nanoparticles/nanostructures at the surface. Thus, powders of detonation nano-diamonds have already found a variety of critical applications in low-energy neutron physics; the range of their applications continues to expand. 

In order to further improve performance of diamond nanoparticle reflectors for slow neutrons, one needs to investigate in detail the following questions: degree of isotopic substitution of hydrogen by deuterium; low-temperature behavior of neutron-nanoparticle inelastic cross-section; possibility of selecting optimum nanoparticle sizes; behavior of nanoparticles in high radiation fluxes. On the other hand, neutrons provide the best method to study hydrogen on surface of diamond nanoparticles.

## References

[1] De Carli P.J., Jameieson J.C. (1961). Formation of diamond by explosive shock. Science.

[2] Alexenskii A.E., Baidakova M.V., Vul A.Y., Siklitskii V.I. (1999). The structure of diamond nanoclusters. Phys. Solid State.

[3] Dolmatov V.Y. (2007). Detonation synthesis nanodiamonds: Synthesis, structure, properties and applications. Rus. Chem. Rev..

[4] Fermi E., Marshall L. (1947). Interference phenomena of slow neutrons. Phys. Rev..

[5] Luschikov V.I., Pokotilovsky Y.N., Strelkov A.V., Shapiro F.L. (1969). Observation of ultracold neutrons. JETP. Lett..

[6] Ignatovich V.K. (1990). The Physics of Ultracold Neutrons.

[7] Golub R., Richardson D.J., Lamoreaux S. (1991). Ultracold Neutrons.

[8] Altarev I.S., Borisov Y.V., Borovikova N.V., Ivanov S.N., Kolomensky E.A., Lasakov M.S., Lobashev V.M., Nazarenko V.A., Pirozhkov A.N., Serebrov A.P. (1992). New measurement of the electric dipole moment of the neutron. Phys. Lett. B.

[9] Baker C.A., Doyle D.D., Geltenbort P., Green K., van der Grinten M.G.D., Harris P.G., Iaydjiev P., Ivanov S.N., May D.G.R., Pendlebury J.M. (2006). Improved experimental limit on the electric dipole moment of the neutron. Phys. Rev. Lett..

[10] Borisov Yu.V., Borovikova N.V., Vasilev A.V., Grigorieva L.A., Ivanov S.N., Kashukeev N.T., Nesvizhevsky V.V., Serebrov A.P., Iaydjiev P. (1988). Study of potential application of ultracold neutrons for measuring the neutron electric charge. J. Tech. Phys..

[11] Nesvizhevsky V.V., Boerner H.G., Petukhov A.K., Abele H., Baessler S., Ruess F.J., Stoeferle Th., Westphal A., Gagarsky A.M., Petrov A.G., Strelkov A.P. (2002). Quantum states of neutrons in the Earth’s gravitational field. Nature.

[12] Mampe W., Ageron P., Bates J.C., Pendlebury J.M., Steyerl A. (1989). Neutron lifetime measured with stored ultracold neutrons. Phys. Rev. Lett..

[13] Nesvizhevsky V.V., Serebrov A.P., Tal’daev R.R., Kharitonov A.G., Alfimenkov V.P., Strelkov A.V., Shvetsov V.N. (1992). Measurement of the neutron lifetime in a gravitational trap and analysis of experimental errors. JETP. Lett..

[14] Mampe W., Bondarenko L.N., Morozov V.I., Panin Yu.N., Fomin A.I. (1993). Measuring neutron lifetime by storing ultracold neutrons and detecting inelastically scattered neutrons. JETP. Lett..

[15] Arzumanov S., Bondarenko L., Chernavsky S., Drexel W., Fomin A., Geltenbort P., Morozov V., Panin Yu., Pendlebury J., Schreckenbach K. (2000). Neutron lifetime value measured by storing ultracold neutrons with detection of inelastically scattered neutrons. Phys. Lett. B.

[16] Pichlmaier A., Butterworth J., Geltenbort P., Nagel H., Nesvizhevsky V.V., Neumaier S., Schreckenbach K., Steichele S., Varlamov V. (2000). MAMBO-II: Neutron lifetime measurement with storage of ultracold neutrons. NIM.

[17] Serebrov A.P., Varlamov V.E., Kharitonov A.G., Fomin A.K., Pokotilovski Yu.N., Geltenbort P., Krasnoschekova I.A., Lasakov M.S., Tal’daev R.R., Vassiljev A.S. (2008). Neutron lifetime measurements using gravitationally trapped ultracold neutrons. Phys. Rev. C.

[18] Nesvizhevsky V.V. (2002). Interaction of neutrons with nanoparticles. Phys. At. Nucl..

[19] Lychagin E.V., Muzychka A.Yu., Nesvizhevsky V.V., Nekhaev G.V., Pignol G., Protasov K.V., Strelkov A.V. (2009). Coherent scattering of slow neutrons at nanoparticles in particle physics experiments. Nucl. Instrum. Methods Phys. Res. A.

[20] Steyerl A., Nagel H., Schreiber F.X., Steinhauser K.A., Gaehler R., Glaeser W. (1986). A new source of cold and ultracold neutrons. Phys. Lett. A.

[21] Altarev I.S., Borovikova N.V., Bulkin A.P., Vesna V.A., Garusov E.A., Grigorieva L.A., Egorov A.I., Erosolimskii B.G., Erykalov A.N., Zakharov A.A. (1986). Universal liquid-hydrogen source of polarized cold and ultracold neutrons at the VVR-M reactor of the Leningrad Institute of nuclear physics. JETP Lett..

[22] Serebrov A.P., Mityukhlyaev V.A., Zakharov A.A., Nesvizhevsky V.V., Kharitonov A.G. (1994). Is it possible to produce next generation of UCN sources with a density 10^3^–10^4^ cm^-3^?. Acta Phys. Hung..

[23] Serebrov A.P., Mityukhlyaev V.A., Zakharov A.A., Kharitonov A.G., Nesvizhevsky V.V., Lasakov M.S., Tal’daev R.R, Alduschenkov A.V., Varlamov V.E., Vasyliev A.V. (1995). Experimental study of a solid-deuterium source of ultracold neutrons. JETP Lett..

[24] Bagrjanov B.V., Kartashov D.G., Kuvshinov M.I., Muzychka A.Y., Nekhaev G.V., Rogov A.D., Smirnov I.G., Stoica A.D., Strelkov A.V., Shvetsov V.N. (1999). Testing experimentally the dynamical-converter method for ultracold neutrons at a pulsed reactor BIGR. Phys. At. Nucl..

[25] Trinks U., Hartmann F.J, Paul S, Schott W. (2000). Concepts of UCN sources for the FRM-II. Nucl. Instrum. Methods Phys. Res. A.

[26] Saunders A., Anaya J.M., Bowles T.J., Filippone B.W., Geltenbort P., Hill R.E., Hino M., Hoedl S., Hogan G.E., Ito T.M. (2004). Demonstration of a solid deuterium source of ultracold neutrons. Phys. Lett. B.

[27] Pokotilovski Yu.N. (1995). Production and storage of ultracold neutrons at pulsed neutron sources with low repetition rates. Nucl. Instrum. Methods Phys. Res. A.

[28] Bondoux D., Boerner H.G., Ermilov V., Gonzales G.P., Kulagin E., Kulikov S., Lelievre-Berna E., Melikhov V., Nesvizhevsky V.V., Soldner T. (2009). Investigation of the energy accumulation rate in solid deuterium irradiated with fast electrons. Nucl. Instrum. Methods Phys. Res. A.

[29] Golub R., Pendlebury J.M. (1975). Super-thermal sources of ultra-cold neutrons. Phys. Lett. A.

[30] Baker C.A., Balashov S.N., Butterworth J., Geltenbort P., Green K., Harris P.G., van der Grinten M.G.D, Iaydjiev P.S., Ivanov S.N., Pendlebury J.M. (2003). Experimental measurement of ultracold neutron production in superfluid ^4^He. Phys. Lett. A.

[31] Schmidt-Wellenburg Ph., Andersen K.H., Courtois P., Kreuz M., Mironov S., Nesvizhevsky V.V., Pignol G., Protasov K.V., Soldner T., Vezzu F. (2009). Ultracold-neutron infrastructure for the gravitational spectrometer GRANIT. Nucl. Instrum. Methods Phys. Res. A.

[32] Ignatovich V.K., Lychagin E.V., Nesvizhevsky V.V., Nekhaev G.V., Muzychka A.Yu., Strelkov A.V. (2002). Neutron transportation in a closed vessel. Phys. At. Nucl..

[33] Schmidt-Wellenburg Ph., Barnard J., Geltenbort P., Nesvizhevsky V.V., Plonka Ch., Soldner T., Zimmer O. (2007). Efficient extraction of a collimated ultra-cold neutron beam using diffusive channels. Nucl. Instrum. Methods Phys. Res. A.

[34] Barnard J., Nesvizhevsky V.V. (2008). Analysis of a method for extracting angularly collimated UCNs from a volume without losing density inside. Nucl. Instrum. Methods Phys. Res. A.

[35] Abele H., Astruc Hoffmann M., Baessler S., Dubbers D., Gluck F., Muller U., Nesvizhevsky V.V., Reich J., Zimmer O. (2002). Is the unitarity of the quark-mixing CKM matrix violated in the neutron β-decay?. Phys. Rev. Lett..

[36] Schumann M., Soldner T., Diessenroth M., Gluck F., Krempel J., Kreuz M., Markisch B., Mund D., Petukhov A., Abele H. (2007). Measurement of the neutrino asymmetry parameter B in neutron beta decay. Phys. Rev. Lett. B.

[37] Vesna V., Gledenov Yu.M., Nesvizhevsky V.V., Petukhov A.K., Sedyshev P.V., Soldner T., Zimmer O., Shulgina E.V. (2008). Measurement of the parity-violating triton emission asymmetry in the reaction ^6^Li(n,α)^3^H with polarized cold neutrons. Phys. Rev. C.

[38] Soldner T., Beck L., Plonka C., Schreckenbach K., Zimmer O. (2004). New limit on T violation in neutron decay. Phys. Lett. B.

[39] Baumann J., Gaehler R., Kalus J., Mampe W. (1998). Experimental limit for the charge of the free neutron. Phys. Rev. D.

[40] Nesvizhevsky V.V., Pignol G., Protasov K.V. (2007). Nanoparticles as a possible moderator for an ultracold neutron source. Int. J. Nanosci..

[41] Nesvizhevsky V.V., Voronin A.Yu., Cubitt R., Protasov K.V. (2009). Neutron whispering gallery. Nat. Phys..

[42] Sheng P. (1990). Scattering and Localization of Classical Waves in Random Media.

[43] Ishimaru A. (1999). Wave Propagation and Scattering in Random Media.

[44] Steyerl A., Trustedt W.D. (1974). Experiments with a neutron bottle. Z. Phys..

[45] Arzumanov S.S., Bondarenko L.N., Geltenbort P., Morozov V.I., Panin Yu.N. (2005). Cold-neutron storage owing to diffusion reflection. Phys. At. Nucl..

[46] Nesvizhevsky V.V., Lychagin E.V., Muzychka A.Yu., Strelkov A.V., Pignol G., Protasov K.V. (2008). The reflection of very cold neutrons from diamond powder nanoparticles. Nucl. Instrum. Methods Phys. Res. A.

[47] Fermi E. (1965). A Course in Neutron Physics.

[48] Artemiev V.A. (2006). Estimation of neutron reflection from nanodispersed materials. At. Energy.

[49] Lychagin E.V., Muzychka A.Yu., Nesvizhevsky V.V., Pignol G., Protasov K.V., Strelkov A.V. (2009). Storage of very cold neutrons in a trap with nano-structured walls. Phys. Lett. B.

[50] Maruyama R., Yamazaki D., Ebisawa T., Hino M., Soyama K. (2007). Development of neutron supermirrors with large critical angle. Thin Solid Films.

[51] Remizovich V.S. (1984). Theory of elastic scattering of particles (photons) incident at grazing angles without application of the diffusion approximation. JETP.

[52] Cubitt R., Lychagin E.V., Muzychka A.Yu., Nekhaev G.V., Nesvizhevsky V.V., Pignol G., Protasov K.V. (2010). Quasi-specular reflection of cold neutrons from nano-dispersed media at above-critical angles. Nucl. Instrum. Method..

[53] Nesvizhevsky V.V., Strelkov A.V., Geltenbort P., Iaydjiev P.S. (1999). Investigation of storage of ultracold neutrons in traps. Europ. J. Appl. Phys..

[54] Nesvizhevsky V.V., Strelkov A.V., Geltenbort P., Iaydjiev P.S. (1999). Observation of new mechanism of ultracold neutron losses in traps. Phys. At. Nucl..

[55] Bondarenko L., Korobkina E., Morozov V., Panin Yu., Geltenbort P., Steyerl A. (1998). Ultracold neutrons cooling during their long dwelling in a trap. JETP Lett..

[56] Bondarenko L.N., Geltenbort P., Korobkina E.V., Morozov V.I., Panin Yu.N. (2002). Cooling and heating of ultracold neutrons during storage. Phys. Atom. Nucl..

[57] Lychagin E.V., Muzychka A.Yu., Nesvizhevsky V.V., Nekhaev G.V., Tal’daev R.R., Strelkov A.V. (2000). Temperature dependence of inelastic ultracold neutron scattering at low-energy transfer. Phys. At. Nucl..

[58] Serebrov A.P., Butterworth J., Daum M., Fomin A.K., Geltenbort P., Kirch K., Krasnoschekova I.A., Lasakov M.S., Rudnev Yu.P., Varlamov V.E. (2003). Low-energy heating of ultracold neutrons during their storage in material bottles. Phys. Lett. A.

[59] Lychagin E.V., Kartashov D.G., Muzychka A.Yu., Nesvizhevsky V.V., Nekhaev G.V., Strelkov A.V. (2002). Mechanism of small variations in energy of ultracold neutrons interacting with a surface. Phys. At. Nucl..

[60] Steyerl A., Yerozolimsky B.G., Serebrov A.P., Geltenbort P., Achiwa N., Pokotilovski Yu.N., Kwon O., Lasakov M.S., Krasnoschekova I.A., Vasilyev A.V. (2002). Experimental study of quasi-elastic scattering of ultracold neutrons. Eur. Phys. J. B.

[61] Strelkov A.V., Nesvizhevsky V.V., Geltenbort P., Kartashov D.G., Kharitonov A.G., Lychagin E.V., Muzychka A.Yu., Pendlebury J.M., Schreckenbach K., Shvetsov V.N. (2000). Identification of a new escape channel for UCN in traps. Nucl. Instrum. Methods Phys. Res. A.

[62] Nesvizhevsky V.V., Lychagin E.V., Muzychka A.Yu., Nekhaev G.V., Strelkov A.V. (2000). About interpretation of experiments on small increase in energy of UCN in traps. Phys. Lett. B.

[63] Nesvizhevsky V.V. (2003). Quantum states of neutrons in a gravitational field and the interaction of neutrons with nanoparticles. Phys. Usp..

[64] Barabanov A.L., Belyaev S.T. (2000). Multiple scattering theory for slow neutrons (from thermal to ultracold). Eur. Phys. J. B.

[65] Ignatovich V.K., and Utsuro M. (2000). Quantum mechanics of the de Broglie wave packet and a review of inelastic losses of UCN in bottles. Nucl. Instrum. Methods Phys. Res. A.

[66] Kartashov D.G., Lychagin E.V., Muzychka A.Yu., Nesvizhevsky V.V., Nekhaev G.V., Strelkov A.V. (2007). An investigation into the origin of small energy changes (~10^-7^ eV) of ultracold neutrons in traps. Int. J. Nanosci..

[67] Atchison F., Brys T., Daum M., Fierlinger P., Geltenbort P., Henneck R., Heule S., Kasprzak M., Kirch K., Pichlmaier A. (2005). First storage of ultracold neutrons using foils coated with diamond-like carbon. Phys. Lett. B.

[68] Brys T., Daum M., Fierlinger P., Foelske A., Gupta M., Henneck R., Heule S., Kasprzak M., Kirch K., Kuzniak M. (2006). Diamond-like carbon coatings for ultracold neutron applications. Diamond Rel. Mat..

[69] Atchison F., Blau B., Daum M., Fierlinger P., Foelske A., Geltenbort P., Gupta M., Henneck R., Heule S., Kasprzak M. (2006). Diamondlike carbon can replace beryllium in physics with ultracold neutrons. Phys. Lett. B.

[70] Atchison F., Blau B., Daum M., Fierlinger P., Geltenbort P., Henneck R., Heule S., Kasprzak M., Kirch K., Kohlik K. (2006). Storage of ultracold neutrons in a volume coated with diamondlike carbon. Phys. Rev. C.

[71] Atchison F., Brys T., Daum M., Fierlinger P., Foelske A., Gupta M., Henneck R., Heule S., Kasprzak M., Kirch K. (2007). Structural characterization of diamond-like carbon films for ultracold neutron applications. Diamond Rel. Mat..

[72] Heule S., Atchison F., Daum M., Foelske A., Henneck R., Kasprzak M., Kirch K., Knecht A., Kuzniak M., Lipper T. (2007). Diamond-like carbon coated ultracold neutron guides. Appl. Surf. Sci..

[73] Atchison F., Blau B., Daum M., Fierlinger P., Geltenbort P., Gupta M., Henneck R., Heule S., Kasprzak M., Knecht A. (2007). Measurement of the Fermi potential of diamond-like carbon and other materials. Nucl. Instrum. Methods Phys. Res. B.

[74] Atchison F., Brys T., Daum M., Fierlinger P., Geltenbort P., Henneck R., Heule S., Kasprzak M., Kirch K., Pichlmaier A. (2007). Loss and spinflip probabilities for ultracold neutrons interacting with diamondlike carbon and beryllium surfaces. Phys. Rev. C.

[75] Atchison F., Bergmaier A., Daum M., Dobeli M., Dillinger G., Fierlinger P., Foelske A., Henneck R., Heule S., Kasprzak M. (2008). Surface characterization of diamond-like carbon for ultracold neutron storage. Nucl. Instrum. Methods Phys. Res. A.

[76] Nesvizhevsky V.V., Pignol G., Protasov K.V., Quemener G., Forest D., Ganau P., Mackowski J.M., Michel Ch., Montorio J.L., Morgado N. (2007). Comparison of specularly reflecting mirrors for GRANIT. Nucl. Instrum. Methods Phys. Res. A.

[77] Krueger A., Kataoka F., Ozawa M., Fujino T., Suzuki Y., Aleksenskii A.E., Vul’ A.Ya., Osawa E. (2005). Usually tight aggregation in detonation nanodiamond: identification and disintegration. Carbon.

[78] Osawa E. (2008). Monodisperse single nanodiamond particles. Pure Appl. Chem..

[79] Avdeev M.V., Rozhkova N.N., Aksenov V.L., Garamuz V.M., Willumeit R., Osawa E. (2009). Aggregate structure in concentrated liquid dispersions of ultrananocrystalline diamond by small-angle neutron scattering. J. Phys. Chem. C.

